# Impact of MRI Texture Analysis on Complication Rate in MRI-Guided Liver Biopsies

**DOI:** 10.1007/s10278-025-01439-0

**Published:** 2025-02-11

**Authors:** Jakob Leonhardi, Maike Niebur, Anne-Kathrin Höhn, Sebastian Ebel, Manuel Florian Struck, Hans-Michael Tautenhahn, Daniel Seehofer, Silke Zimmermann, Timm Denecke, Hans-Jonas Meyer

**Affiliations:** 1 Department of Diagnostic and Interventional Radiology, University Hospital Leipzig, Liebigstraße 20, 04103 Leipzig, Germany; 2 Department of Pathology, University Hospital Leipzig, Leipzig, Germany; 3 Department of Anesthesiology and Intensive Care Medicine, University Hospital Leipzig, Leipzig, Germany; 4 Department of Visceral, Thoracic and Vascular Surgery, University Hospital Leipzig, Leipzig, Germany; 5 Institute of Laboratory Medicine, Clinical Chemistry and Molecular Diagnostics, University Hospital Leipzig, Leipzig, Germany

**Keywords:** MRI, Liver biopsy, Hemorrhage, Radiomics, Texture analysis

## Abstract

Magnetic resonance imaging (MRI)–derived texture features are quantitative imaging parameters that may have valuable associations with clinical aspects. Their prognostic ability in patients undergoing percutaneous MRI-guided liver biopsy to identify associations with post-interventional bleeding complications and biopsy success rate has not been sufficiently investigated. The patient sample consisted 79 patients (32 females, 40.5%) with a mean age of 58.7 ± 12.4 years. Clinical parameters evaluated included comorbidities, pre-existing liver disease, known cancer diagnosis, and hemostaseological parameters. Several puncture-related parameters such as biopsy angle, distance of needle entry to capsule, and lesion were analyzed. MRI texture features of the target lesion were extracted from the planning sequence of the MRI-guided liver biopsy. Mann–Whitney *U* test and Fisher’s exact test were used for group comparison; multivariate regression model was used for outcome prediction. Overall, the diagnostic outcome of biopsy was malignant in 38 cases (48.1%) and benign in 32 cases (40.5%). A total of 11 patients (13.9%) had post-interventional bleeding, while nine patients (11.4%) had a negative biopsy result. Several texture features were statistically significantly different between patients with and without hemorrhage. The texture feature GrVariance (1.37 ± 0.78 vs. 0.80 ± 0.35, *p* = 0.007) reached the highest statistical significance. Regarding unsuccessful biopsy results, S(1,1)DifEntrp (0.80 ± 0.10 vs. 0.89 ± 0.12, *p* = 0.022) and S(0,4)DifEntrp (1.14 ± 0.10 vs. 1.22 ± 0.11, *p* = 0.021) reached statistical significance between groups. Several MRI texture features of the target lesion were associated with bleeding complications or negative biopsy after MRI-guided percutaneous liver biopsy. This could be used to identify at-risk patients at the beginning of the procedure and should be further analyzed.

## Background

Unclear focal lesions of the liver can be clinically challenging and include metastases, primary tumors with the need for a correct diagnosis [1–3].

Cross-sectional imaging with computed tomography (CT) and magnetic resonance imaging (MRI) are the methods of choice to detect and characterize liver lesions, but in some cases, histopathological conformation is still required [2, 4]. In particular, small lesions with indistinct contrast media enhancement can impose a challenging diagnosis and should be further secured by biopsy. In contrast, some lesions such as a hepatocellular carcinoma in a cirrhotic liver with typical contrast media enhancement can be diagnosed non-invasively [3, 4].

Image-guided percutaneous biopsy is the established method for tissue sampling in clinical routine [5]. In most centers, ultrasound and CT guidance are used for liver biopsy [6]. CT guidance allows a better visualization of the needle tip and may provide more overview of anatomical structures during the procedure [7].

The best visualization of a focal liver lesion can be achieved with MRI using liver-specific contrast agents [8–11]. Early studies using this imaging modality reported a high accuracy with overall 96%, resulting in a sensitivity of 95.5% and specificity of 100.0%, respectively [8–10]. Other authors even reported a success in every case [9]. However, other studies demonstrated worse outcomes with a diagnostic yield of 61%, which was inferior to CT-guided biopsies [12].

The overall complication rate of liver biopsies has been reported to be 0.4–1.7% with an estimated procedure-specific mortality of 0.11% [6, 13–15]. Typical complications of the procedure comprise severe pain, hypotension, bleeding, fluid collections, and pneumothorax [13].

A large representative study demonstrated a complication rate of 1.6% in a total of 2405 liver biopsies with sonography guidance [14]. Of these, a total of 38 patients (1.6%) had postinterventional bleeding with hemoperitoneum or liver hematoma, respectively [14]. Therefore, the identification of patients at risk for postbioptic complications is of great importance.

Texture analysis can be used to provide quantitative data from radiological images [16–20]. This method reflects the microstructural characteristics of tissues with imaging modalities [20–22]. This may particular be important as the MRI sequences used to plan the MRI-guided biopsy may have diagnostic and prognostic relevance for the ongoing biopsy.

Presumably, MRI texture analysis characterizes the target lesions of patients at risk for bleeding complications and with this approach better stratify patients at risk for complications of MRI-guided liver biopsy. This could change the clinical course of the patient, when the postinterventional bleeding risk is too high.

Therefore, the present analysis used quantitative imaging features to predict postinterventional bleeding complications and negative biopsy outcome in patients undergoing percutaneous MRI-guided liver biopsy.

## Methods

### Patient Acquisition

The present retrospective study received approval from the local ethics committee of the University Hospital of Leipzig (register no. 344-2007).

All consecutive patients undergoing MRI-guided liver biopsy at the University Hospital of Leipzig between the years 2012 and 2021 were analyzed in this observational study. Written informed consent was obtained from all patients before MRI-guided biopsy.

In our center, MRI-guided biopsy is mainly used for small target lesions with poor visibility on CT images.

### MRI-Guided Biopsy

All MRI-guided liver biopsies were performed by trained interventional radiologists with at least 2 years of general experience in interventional radiology. The procedure plan (comprising the position and the needle pathway) was calculated using previous cross-sectional images. The length and angle of the biopsy needle were measured based on the planning scan.

All biopsies were performed in a conventional closed-bore 1.5-T MRI scanner (Magnetom Symphony and Aera, Siemens Health care, Erlangen, Germany) with a bore diameter of 60 cm and a gantry length of 150 cm.

For procedure planning, transverse images were acquired with volume-interpolated breath-hold examination (VIBE), half-Fourier single-shot turbo spin echo (HASTE) using a combination of loop and spine coils. In all cases, 10 mL of the liver-specific contrast agent gadoxetate disodium (Primovist®, Bayer HealthCare Pharmaceuticals, Berlin, Germany) was administered intravenously approximately 15 min prior to the intervention to provide a negative contrast in the VIBE sequence. The same sequence was used during the time interval of the study.

The target lesion was measured on the planning VIBE on the representative slice with the largest diameter. A biopsy tract was selected on the planning sequence to avoid penetrating large vessels.

All biopsies were performed after confirmation of the absence of coagulation disorders (platelet count of at least 50,000/mm^3^, activated partial thromboplastin time [aPTT] ≤ 50 s, and prothrombin time [Quick] > 50%). The procedure started with skin disinfection and local anesthesia with 10 mL lidocaine 1% (Xylocitin, Jenapharm, Germany). During the procedure, T1-weighted images were used for needle visualization and placement within the target lesion. In every case, multiple tissue samples were obtained with the biopsy system (18G semiautomatic biopsy gun, Invivo, Imaging Solutions, Shailer Park, Australia).

After removal of the biopsy needle, T1- and T2-weighted images covering the whole liver were acquired to rule out post-interventional bleeding complications.

Every form of novel fluid surrounding the liver after the intervention was defined as hematoma. The hematoma width was measured on one axial slice using the perpendicular diameter.

The diagnostic outcome of the biopsy was determined by the pathology report and with the need of re-biopsy during the clinical course of the patient.

### Biopsy-Related Parameters

Several parameters were measured as biopsy-related features on the axial MRI slices: the distance of needle entry to liver capsule, the distance of the lesion to the liver capsule, the distance of the lesion to the needle entry of the liver, the needle angle of the entry. The maximum size of the target lesion was measured with two perpendicular diameters on the largest, representative slice. Finally, the time duration of the biopsy was defined between the beginning of the planning scan to the last control sequence after the needle removal.

### MRI Texture Analysis

MRI images were further analyzed with the dedicated software MaZda (version 4.7, available at http://www.eletel.p.lodz.pl/mazda/) [23, 24]. Texture analysis measurements were blindly carried out to the bioptic and clinical results. All measurements were performed by a trained radiologist with overall 4 years of experience. A region of interest (ROI) was placed on the largest slice of the target lesion using the T1-weighted VIBE sequence for biopsy planning after application of hepatic specific contrast media. The ROI was drawn within the liver lesion with 2 mm distance to the adjacent parenchyma. For each ROI, gray-level (µ) normalization was utilized to μ ± 3 standard deviations to reduce the influence of contrast and brightness variation on the texture features, as performed previously [15, 16]. Texture features of various groups were extracted for each patient including histogram parameters, second-order texture features of different groups comprising (co-occurrence matrix run-length matrix, absolute gradient, autoregressive model [theta 1 to 4, sigma], and wavelet transform features).

Altogether, 279 texture features were calculated in all lesions. A correlation analysis was performed to remove redundant texture features with a correlation coefficient larger than 0.7.

**Fig. 1:**
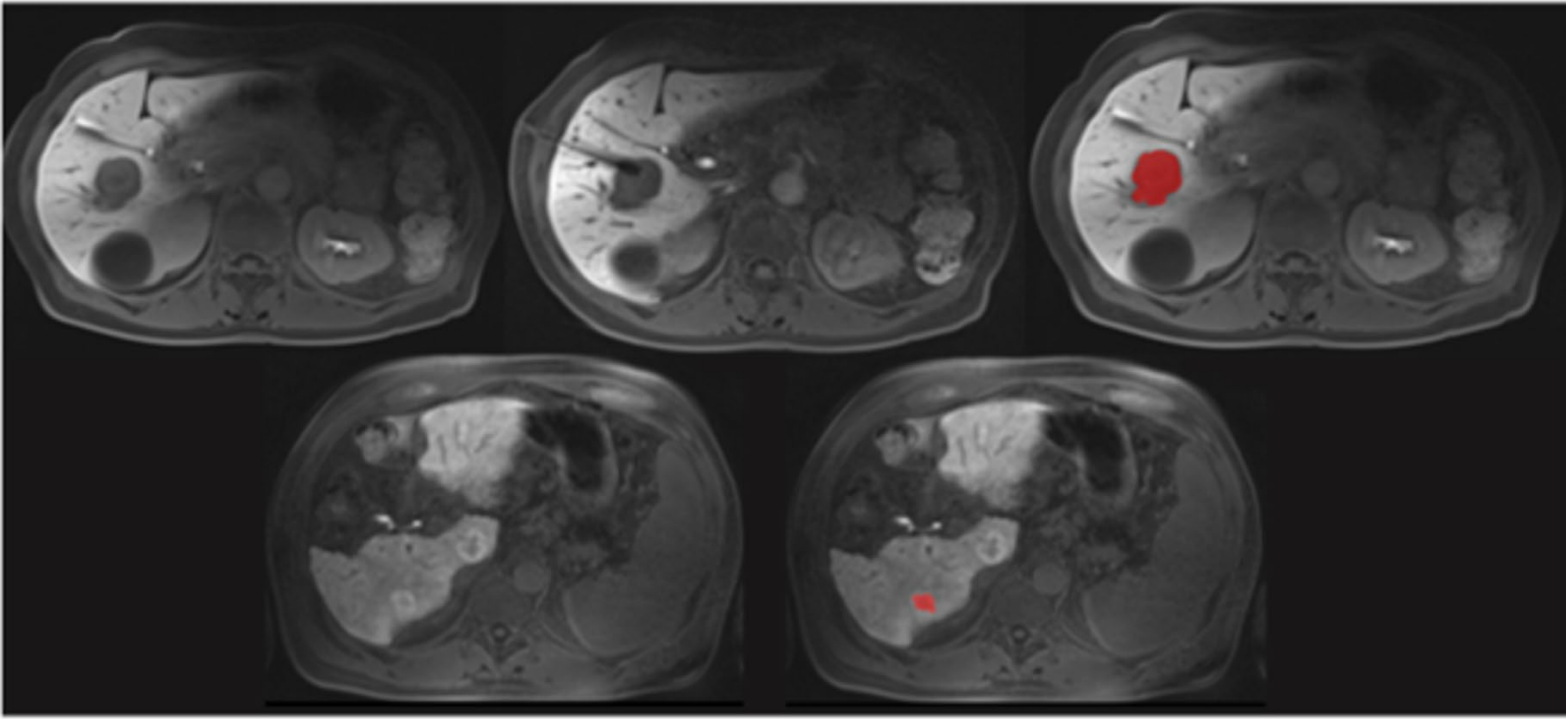
Representative cases of the patient sample. First column: 51-year-old female patient with a diagnostic biopsy of a lesion in segment V confirmed as a metastasis of a cervical carcinoma. The biopsy needle is shown within the needle. The corresponding region of interest is marked in red. Second column: 59-year-old male patient with two unclear tumors with central hypointense signal intensity and peripherally contrast media uptake in liver segments VI and I. Slight intrahepatic cholestasis, steatosis degree 15%, moderate fibrosis, and diagnostic biopsy in segment VI with a benign outcome of the biopsy. No postinterventional hemorrhage. The corresponding region of interest is marked in red

Two representative cases of the patient cohort are shown in Fig. 1 for illustrative purposes.

### Statistical Analysis

Statistical analysis was performed with SPSS (IBM, Version 25.0; Armonk, NY, USA). Collected data was primarily assessed with descriptive statistics. Group differences were analyzed with Mann–Whitney *U* test and Fisher’s exact test when suitable. Receiver operating characteristics (ROC) curve with area under the curve (AUC) analyses was used to investigate the diagnostic accuracy for the clinical and MRI texture parameters. Univariate and multivariate logistic regression analyses were performed to further elucidate the associations between MRI texture features with the investigated biopsy outcomes. A multivariate model was built using the statistically significant texture features.

In all instances, *p*-values below 0.05 were considered as statistically significant.

## Results

The patient sample comprised 79 patients (32 females, 40.5%) with a mean age of 58.7 ± 12.4 years. In 70 cases (88.6%), the biopsies were performed successfully with a diagnostic outcome.

Overall, 38 cases were malignant (48.1%) and 32 were benign (40.5%).

For the malignant cases, hepatocellular carcinoma was diagnosed in 13 cases (16.5%), 15 cases with metastases of different primary tumors (19.0%), six cases (7.6%) with cholangiocarcinoma, followed by one case (1.3%) with a sarcoma and lymphoma, respectively.

### Postinterventional Hemorrhage

A total of 11 patients (13.9%) had postinterventional hemorrhage. Mean hemorrhage width was 6.6 mm ± 3.5 mm. In one case, hemorrhage resulted in the necessity of gelfoam-application via the puncture needle to stop the bleeding. No further emergency treatment was needed, and no fatal outcome occurred in this patient cohort.

A discrimination analysis between the patient groups with hemorrhage and without was carried out. The investigated clinical and puncture-related features are shown in Tables 1 and 2. No statistically significant differences were identified between both groups regarding the clinical and puncture-related features.

**Table 1:** The investigated clinical features in comparison between cases with postinterventional hemorrhage and without hemorrhage

Clinical and laboratory features	Hemorrhage (*n* = 11)	No hemorrhage (*n* = 68)	*p*-value
Gender (female)	6 (54.4%)	26 (38.2%)	0.31
Age (years)	59.91 ± 9.48	58.77 ± 12.57	0.99
Prior cancer diagnosis	8 (72.7%)	32 (47.1%)	0.12
Malignant pathology	5 (45.4%)	33 (48.5%)	0.94
Cholestasis	1 (9.1%)	14 (20.6%)	0.37
Steatosis degree (%)	0.10 ± 0.12	0.20 ± 0.18	0.09
Fibrosis	9 (81.8%)	56 (82.4%)	0.44
Diabetes mellitus	5 (45.4%)	19 (27.9%)	0.25
Arterial hypertension	7 (63.6%)	43 (63.2%)	0.91
aPTT (s)	28.83 ± 2.82	30.23 ± 3.93	0.21
Platelet count in 10^3^/µL	231.27 ± 97.76	179.74 ± 88.34	0.20
Quick (%)	95.73 ± 14.51	94.13 ± 17.62	0.54

**Table 2:** The investigated puncture-related features in comparison between cases with and without postinterventional hemorrhage

Puncture-related features	Hemorrhage (*n* = 11)	No hemorrhage (*n* = 68)	*p*-value
Distance of needle entry to liver capsule (mm)	24.6 ± 12.9	23.3 ± 13.0	0.52
Distance lesion from liver capsule (mm)	5.9 ± 8.6	7.5 ± 9.6	0.64
Distance of lesion to needle entry (mm)	44.4 ± 18.2	49.8 ± 29.6	0.65
Largest lesion diameter (mm)	23.3 ± 19.8	27.9 ± 22.2	0.42
Needle angle (°)	62.5 ± 19.4	62.8 ± 18.7	0.97
Duration of intervention (minutes)	17.8 ± 11.8	14.2 ± 7.8	0.35
Success rate of biopsy	9 (81.8%)	61 (89.7%)	0.45

### MRI Texture Features

In the next step, the differences in regard of the MRI texture features were investigated. The results of the discrimination analysis are presented in Table 3. The following texture features demonstrated the best results to discriminate cases with hemorrhage versus non-hemorrhage: S(1,0)DifVarnc (5.82 ± 5.34 vs. 2.72 ± 1.55, *p* = 0.015), S(1,−1)DifVarnc (9.35 ± 8.29 vs. 4.46 ± 2.49, *p* = 0.010), GrVariance (1.37 ± 0.78 vs. 0.80 ± 0.35, *p* = 0.007), and GrSkewness (0.91 ± 0.76 vs. 0.33 ± 0.40, *p* = 0.012).

**Table 3:** An overview of the statistically significant texture features in comparison between cases with postinterventional hemorrhage and those without hemorrhage. The features in bold letters were added to the multivariate model prediction of hemorrhage

Texture features	Hemorrhage (*n* = 11)	No hemorrhage (*n* = 68)	*p*-value
_MinNorm	65.91 ± 26.61	52.15 ± 20.88	0.041
Perc,01%	79.73 ± 25.57	65.31 ± 20.83	0.034
Perc,10%	85.64 ± 25.70	71.46 ± 21.73	0.047
S(1,0)Contrast	11.27 ± 10.85	5.98 ± 3.43	0.036
S(1,0)Correlat	0.94 ± 0.06	0.97 ± 0.02	0.029
**S(1,0)DifVarnc**	**5.82 ± 5.34**	**2.72 ± 1.55**	**0.015**
S(0,1)Contrast	9.05 ± 7.30	5.55 ± 3.32	0.049
S(0,1)DifVarnc	4.21 ± 3.19	2.40 ± 1.40	0.032
S(1,1)DifVarnc	8.06 ± 6.27	4.51 ± 2.62	0.023
S(1,−1)Contrast	19.01 ± 16.15	10.67 ± 6.02	0.022
**S(1,−1)Correlat**	**0.90 ± 0.10**	**0.95 ± 0.03**	**0.019**
**S(1,−1)DifVarnc**	**9.35 ± 8.29**	**4.46 ± 2.49**	**0.010**
**S(2,0)DifVarnc**	**16.80 ± 13.50**	**8.46 ± 4.82**	**0.017**
S(0,2)DifVarnc	12.57 ± 8.61	7.59 ± 4.47	0.039
S(2,2)DifVarnc	21.79 ± 13.79	13.74 ± 7.69	0.032
S(2,−2)Contrast	54.17 ± 37.18	34.74 ± 19.14	0.035
S(2,−2)Correlat	0.69 ± 0.24	0.82 ± 0.10	0.035
**S(2,−2)DifVarnc**	**24.54 ± 17.19**	**13.62 ± 7.24**	**0.017**
S(3,0)DifVarnc	27.84 ± 19.43	15.69 ± 8.55	0.029
S(3,3)SumVarnc	294.48 ± 63.36	331.55 ± 49.64	0.047
S(3,3)DifVarnc	32.72 ± 14.97	23.78 ± 12.40	0.043
S(3,−3)DifVarnc	37.40 ± 21.04	23.83 ± 11.78	0.020
S(4,0)DifVarnc	35.62 ± 20.22	22.84 ± 11.73	0.044
S(4,4)SumVarnc	268.51 ± 61.61	306.16 ± 54.96	0.046
S(4,−4)DifVarnc	47.14 ± 22.73	32.91 ± 15.18	0.027
**GrVariance**	**1.37 ± 0.78**	**0.80 ± 0.35**	**0.007**
**GrSkewness**	**0.91 ± 0.76**	**0.33 ± 0.40**	**0.012**
**GrKurtosis**	**1.99 ± 3.39**	**0.14 ± 1.32**	**0.016**
WavEnLH_s-1	38.05 ± 29.79	22.77 ± 14.30	0.043
WavEnHL_s-1	47.68 ± 39.05	25.60 ± 15.38	0.025
WavEnHH_s-1	2.16 ± 3.38	0.97 ± 0.89	0.047
WavEnHL_s-2	147.46 ± 110.65	87.53 ± 53.26	0.036
WavEnHH_s-2	15.86 ± 32.37	4.32 ± 4.17	0.023
WavEnHL_s-4	568.30 ± 375.59	366.98 ± 243.93	0.040
WavEnHH_s-4	119.21 ± 68.10	89.10 ± 98.61	0.022

### Multivariate Model to Predict Postinterventional Hemorrhage

**Fig. 2:**
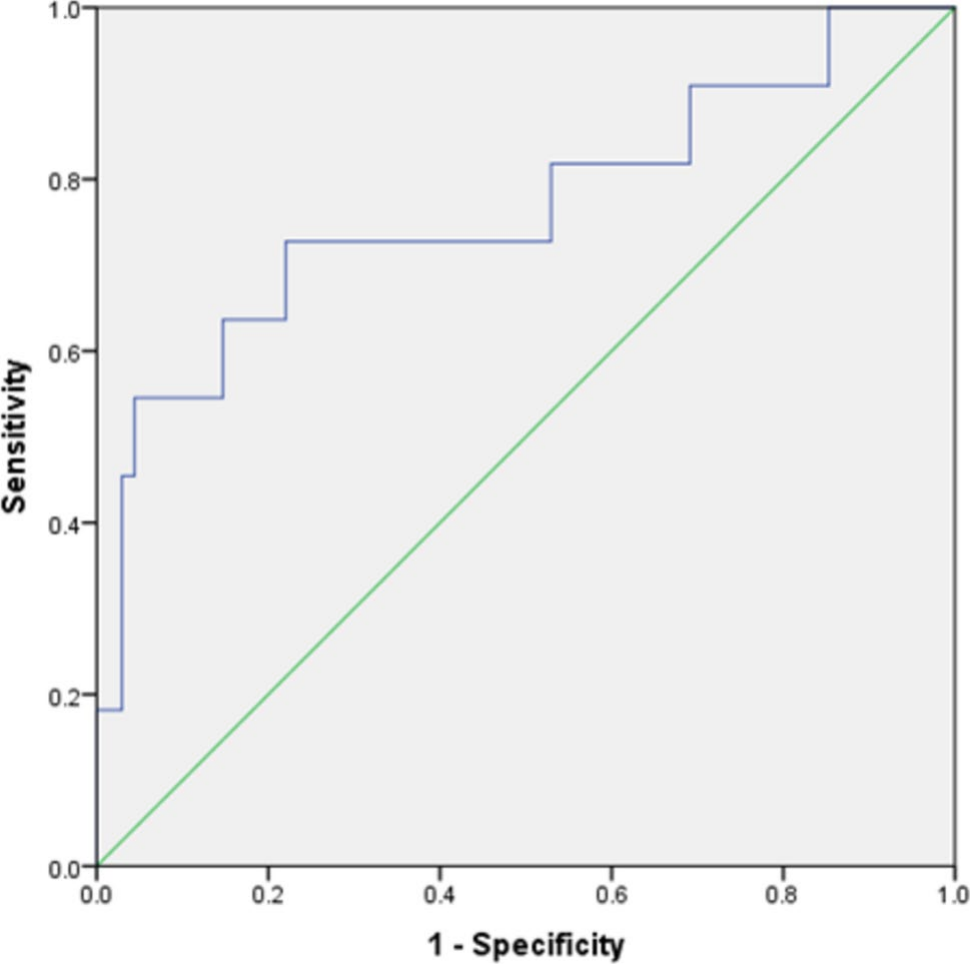
ROC curve of the prediction model for hemorrhage based on MRI texture features, resulting in an area under the curve of 0.77 (95% CI 0.59–0.95)

A multivariate model using eight texture features was built to predict the postinterventional hemorrhage. This model demonstrated a diagnostic accuracy with an AUC of 0.77 [95% CI 0.59–0.95], *p* = 0.005. The resulting sensitivity was 72.7% with a specificity of 77.9%. The resulting graphs are displayed in Fig. 2.

### Success of the Biopsy

A discrimination analysis was carried out between the groups according to the biopsy success. No clinical feature was statistically significant different between both groups (Tables 4 and 5).

**Table 4:** An overview of the investigated clinical features in comparison between cases with diagnostic biopsies and non-diagnostic biopsies

Clinical features	Diagnostic biopsy (n = 70)	Non-diagnostic biopsy (*n* = 9)	*p*-value
Gender (female)	28 (40.0%)	4 (44.4%)	0.80
Age (years)	61.25 ± 9.49	56.96 ± 13.59	0.67
Prior cancer diagnosis	34 (48.6%)	6 (66.7%)	0.31
Cholestasis	15 (21.4%)	0 (0%)	0.13
Steatosis	0.19 ± 0.18	0.17 ± 0.18	0.19
Fibrosis	57 (81.4%)	8 (88.9%)	0.09
Diabetes mellitus	19 (27.1%)	5 (55.6%)	0.09
Arterial hypertension	44 (62.9%)	6 (66.7%)	0.76
aPTT (s)	29.2 ± 3.1	30.5 ± 4.1	0.59
Platelet count	191.9 ± 84.9	190.3 ± 99.7	0.33
Quick (%)	96.3 ± 12.8	92.7 ± 19.9	0.78

**Table 5:** The investigated puncture features in comparison between cases with diagnostic biopsy result and those without

Puncture features	Diagnostic biopsy (*n* = 70)	Non diagnostic biopsy (*n* = 9)	*p*-value
Distance of needle entry to liver capsula (mm)	24.1 ± 13.1	18.4 ± 11.0	0.25
Distance lesion from liver capsula (mm)	7.2 ± 9.3	8.4 ± 10.9	0.77
Distance of lesion to needle entry (mm)	49.3 ± 27.7	47.5 ± 34.7	0.75
Largest lesion diameter (mm)	28.1 ± 22.3	20.6 ± 16.4	0.16
Needle angle (°)	62.1 ± 19.2	68.3 ± 13.3	0.45
Duration of intervention (min)	14.7 ± 8.4	14.7 ± 9.0	0.94

Several MRI texture features showed statistically significant differences between the two groups. These results are presented in Table 6. The texture features S(1,1)DifEntrp (0.80 ± 0.10 vs. 0.89 ± 0.12, *p* = 0.022), S(0,4)DifEntrp (1.14 ± 0.10 vs. 1.22 ± 0.11, *p* = 0.021) showed the highest significance.

**Table 6:** Texture features showing statistically significant differences in comparison between cases with diagnostic biopsy and without diagnostic biopsies

Texture features	Diagnostic biopsy (*n* = 70)	Non-diagnostic biopsy (*n* = 9)	*p*-value
S(0,1)DifEntrp	0.67 ± 0.09	0.76 ± 0.13	0.027
S(1,1)DifEntrp	0.80 ± 0.10	0.89 ± 0.12	0.022
S(0,2)DifEntrp	0.91 ± 0.10	1.00 ± 0.13	0.022
S(2,2)DifEntrp	1.03 ± 0.10	1.12 ± 0.12	0.027
S(2,−2)DifEntrp	1.03 ± 0.10	1.11 ± 0.11	0.048
S(0,3)DifEntrp	1.05 ± 0.10	1.14 ± 0.12	0.022
S(3,3)DifEntrp	1.15 ± 0.10	1.23 ± 0.11	0.037
S(0,4)DifEntrp	1.14 ± 0.10	1.22 ± 0.11	0.021
S(0,5)DifEntrp	1.20 ± 0.10	1.28 ± 0.10	0.023

### Multivariate Model to Predict Successful Biopsy

**Fig. 3:**
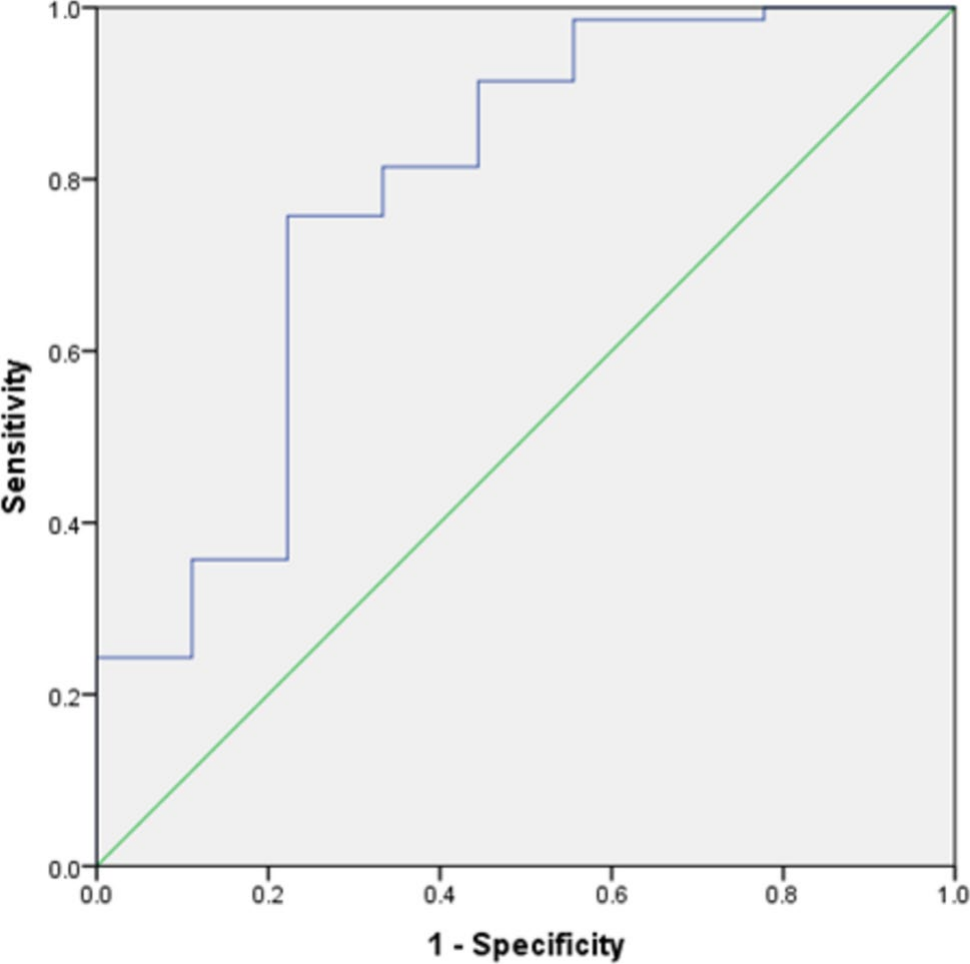
ROC curve of the prediction models of a diagnostic biopsy outcome based on the MRI texture features, resulting in an area under the curve of 0.78 (95% CI 0.60–0.97)

A multivariate model using nine texture features predicted the successful biopsy with an AUC of 0.78 [95% CI 0.60–0.97], *p* = 0.006. The resulting sensitivity was 75.7% with a specificity of 77.8%. The corresponding graph is displayed in Fig. 3.

## Discussion

The present analysis investigated the predictive relevance of MRI-derived texture features in MRI-guided liver biopsy. As a key finding, several features were associated with postinterventional bleeding and unsuccessful biopsy outcome after MRI-guided liver biopsy.

The present study tried to establish possible associations between the target lesion microstructure, provided by the MRI-extracted texture features, with postinterventional complications.

Our identified rate of successful biopsy results is in line with previous studies with an overall good outcome rate of 88.6% [8–12]. The overall diagnostic yield of MRI-guided liver biopsies ranged from 61 to 100% in the published literature [8–12].

Therefore, MRI-guided liver biopsy can be considered as the imaging modality of choice with the best visualization of the target lesion [11]. However, this also comes at the highest biopsy costs with longer procedure time and material costs [12].

Notably, the postinterventional complications of this procedure has not been systematically evaluated in large series due to its clinical indication spectrum compared with sonography- or CT-guided liver biopsies.

Moreover, the present results can provide the potential benefit of texture analysis of the target lesion to identify patients at risk for a negative biopsy result and postinterventional hemorrhage. Several texture features derived from the co-occurrence matrix run-length matrix group were different between patients with and without post-interventional hemorrhage. These features reflect the heterogeneities of the grey levels of the target lesion in a spatial manner [16–19]. Presumably, using this approach, it is possible to better characterize tumors with a higher potential for bleeding complications.

Despite its low-risk profile of percutaneous liver biopsy, several complications have been reported in the literature using different forms of imaging guidance [6, 7, 15]. Among them and most severe, bleeding is associated with a potential lethal outcome. A large older study investigating a total of 9212 sonography-guided liver biopsies reported 11 fatal (0.11%) and 22 nonfatal hemorrhages (0.24%) [15]. Another representative study of 68,276 biopsies between the years 1973 to 1983 reported fatal outcomes in nine of 100,000 cases due to hemorrhage [5]. However, Midia et al. reported bleeding of any kind in up to 10.9% of cases after liver biopsies, which is comparable with the present study [7]. The differences between the studies can be accounted to study criteria and definition of postinterventional hemorrhage. We included every new postinterventional fluid surrounding the liver, regardless of clinical presentation. This might explain our higher frequency of bleedings compared with previous cohorts using ultrasonography for detection of postinterventional symptomatic patients.

One shortcoming of the present results is the only moderate accuracy to predict postinterventional hemorrhage. A better non-invasive characterization of the target lesion and the underlying histopathology may only have a small impact on the bleeding risk of the patient and may be more associated with procedure-related features, which were not considered in the present analysis. However, one should consider that even extensive hemostaseology workup procedures as performed in a recent study from France including several different assays have only a low predictive value [25]. The authors concluded that adding global coagulation assays to the routine tests of platelet count, PTT and prothrombin rate does not improve the prediction of bleeding [25]. 

This point further strengthens the rationale of the present study to identify novel quantitative imaging markers to characterize patients at risk.

The present analysis is not free from limitations. First, it is a retrospective study with known inherent bias. Second, although in all cases a 18-G needle was used for the biopsy, the needle length varied slightly due to the localization of the target lesion. Third, all patients with post-biopsy bleeding were included into the present analysis, independent from clinical presentation. No further subgroup analyses in accordance to clinical symptoms were possible in the current study. Moreover, no further subgroup analyses according to the tumor type could be performed, especially a comparison between primary and secondary liver tumor. There may be significant differences between the groups, which cannot be adjusted for in the present study due to the small patient sample. Fourth, there was no further analysis for the relationship of the target lesion and the surrounding vessels. This could be a confounder for the postinterventional hemorrhage. Fifth, there might be some interreader bias of the ROI placement. However, due to the high contrast of the T1-weighted images, the measurement can be considered as reliable. Sixth, we used the freely available MaZda package for the texture analysis. There may be some differences in regard of the extracted texture features, when compared with other texture packages due to underlying formulas and different extracted features.

## Conclusion

Several MRI texture features were associated with postinterventional hemorrhage and negative biopsy result after MRI-guided percutaneous liver biopsy. These features could be used to identify patients at risk at the beginning of the procedure.

## Data Availability

Data generated or analyzed during the study are available from the corresponding author by request.

## Abbreviations

CT: Computed tomography
MRI: Magnetic resonance imaging
AUC: Area under the curve
ROC: Receiver operating characteristics
PTT: Prothrombin time
